# Chemical Composition of the Uterine Fibres in Pregnancy

**Published:** 1853-03

**Authors:** 


*Chemical Composition of the Uterine Fibres in Pregnancy.* By SIEGMUND.—The point of interest in this communication is, that kreatin was obtained from the tissue of the gravid uterus. Formic and acetic acids were also present.—*British and Foreign Medico-Chirurgical Review,* from *Wurzburg Gesell. Verhandl.*

